# War Surgery in Spain
*(i) Treatment of War Wounds and Fractures. J. Trueta. Hamish Hamilton & Co.(ii) "War Surgery in Spain." Tudor Hart. British Medical Journal, 1939, i., p. 1099.


**Published:** 1939

**Authors:** A. Rendle Short


### WAR SURGERY IN SPAIN.*

ALTHOUGH the anticipated bombing attacks on our towns and seaports have not taken place, everyone realizes that it would be too sanguine to jump to the conclusion that we are free for ever from the menace: and medical practitioners even in the West of England should know what lines of treatment ought to be followed if they find themselves called upon to deal with numerous casualties during or just after a raid. It is curious that experience in one war appears to be almost valueless in the next. Treatment of wounds of the chest and of the abdomen was quite different in the South African War and in the Great War. The methods that proved most valuable in Madrid and Barcelona during the recent fighting in Spain would have been considered revolutionary in 1917 or 1918.

Air-raid casualties fall into three groups. Machine-gun bullet wounds are relatively innocuous, unless the bullet is retained, or a bone is shattered. Small air bombs throw out small splinters of high velocity; they produce insignificant and often painless skin wounds, but the damage to muscles or other internal structures may be very extensive. The patient must be stripped and looked well over, or these may be missed. Most of the casualties, however, are big smashes due to falling masonry or fragments of large bombs, with grave compound fractures or joint injuries. The Spanish surgeons stress the importance of really immediate operation, which ought to be performed under six hours. If there is longer delay serious infections take place in the devitalized tissue and a long convalescence is probable. The corollary is that first-aid treatment must be quick and simple: to stop bleeding; to apply a very temporary splint for purposes of transport, such as a Thomas splint over the clothes for a broken leg; and to give morphia—this is all that time permits. The surgeons at the hospitals in Spain did not wait for the end of the raid, but operated through it. They might even

*(i) *Treatment of War Wounds and Fractures.* J. Trueta. Hamish Hamilton & Co.

(ii) "War Surgery in Spain." Tudor Hart. *British Medical Journal,* 1939, i., p. 1099.

have to wear gas-masks. The purpose of the operation was a thorough débridement, as performed in the later stages of the Great War, to remove all dead or damaged tissue, and to adjust fractures. Bleeding should be stopped by pressure, not by ligatures. Two methods were then available. The wound might be closed forthwith, and no dressing whatever applied. This is only suitable if the case is treated early, the débridement is thorough, the skin comes together with no tension, and the patient can remain for a week under the care of the same surgeon. If any of these conditions are lacking the wound is left open, packed with gauze or vaseline-gauze, and the limb fixed in a plaster case, without any padding between it and the skin. The case is left on for several weeks. It is admitted that the smell becomes vile on account of wound discharges, but the patient is comfortable, and he can be transported to a place of safety. Wounds of joints must, if possible, be closed. In the Great War we made strenuous efforts to close the capsule, but apparently it is just as good to close the skin and leave the capsule unsutured. Fractures may be held in position by Kirschner wires.

The most surprising part of Spanish experience is that even when wounds are suppurating, the best treatment is to encase them in plaster, but only after a thorough drainage operation to open up all the pockets. For a few days the temperature may run high, but this is no indication for removing the plaster. The case is left on for several weeks: changing it usually involves another flare-up of the temperature. Strange to say, gas gangrene very seldom occurs in wounds thus treated. Foreign observers were apt to conclude that Spanish soil does not contain the Bacillus welchii, but this is certainly incorrect; before these methods of treatment were evolved, plenty of cases of gas gangrene were seen. Anti-tetanic serum is valuable, and should be given as early as possible; but Spanish surgeons have their doubts as to the value of anti-gas-gangrene serum.

Limbs should be kept elevated. Nerves and tendons are treated by late, not by primary suture, which is sure to fail. Windows must not be cut in plaster cases; the tissues bulge into the window and may slough. There are certain cases that should *not* be put up in plaster forthwith. These are patients with injury of the main artery to a limb and threatened loss of circulation in the leg; and patients with superficial cellulitis. The latter should be treated by means of multiple incisions, and plastered a few days later when the acute phase is over.

By treatment along these lines, Trueta is able to report that of 1,073 cases of open fracture only 6 died, 976 gave satisfactory result, and 91 a poor result. These last include secondary amputations, and patients with persistent osteitis, or non-union of fractures.

"By rapid, properly planned, boldly executed surgery, followed by closed Plaster of Paris casts, the casualties of war can to-day be spared the torment of having to pass the rest of their days crippled and mutilated."

One must pay a tribute to the heroism of the Barcelona surgeons and their teams, who worked through the strain of 300 air-raids to obtain such wonderful results.

A. RENDLE SHORT.

